# Daratumumab/lenalidomide/dexamethasone in transplant-ineligible newly diagnosed myeloma: MAIA long-term outcomes

**DOI:** 10.1038/s41375-024-02505-2

**Published:** 2025-02-27

**Authors:** Thierry Facon, Philippe Moreau, Katja Weisel, Hartmut Goldschmidt, Saad Z. Usmani, Ajai Chari, Torben Plesner, Robert Z. Orlowski, Nizar Bahlis, Supratik Basu, Cyrille Hulin, Hang Quach, Michael O’Dwyer, Aurore Perrot, Caroline Jacquet, Christopher P. Venner, Noopur Raje, Mourad Tiab, Margaret Macro, Laurent Frenzel, Xavier Leleu, Gordon Cook, George Wang, Huiling Pei, Maria Krevvata, Robin Carson, Fredrik Borgsten, Shaji K. Kumar

**Affiliations:** 1 University of Lille, CHU Lille, Service des Maladies du Sang, Lille, France; 2 Hematology Department, University Hospital Hôtel-Dieu, Nantes, France; 3 Department of Oncology, Hematology and Bone Marrow Transplantation with Section of Pneumology, University Medical Center Hamburg-Eppendorf, Hamburg, Germany; 4 GMMG-Study Group at University Hospital Heidelberg, Internal Medicine V, Heidelberg, Germany; 5 Memorial Sloan Kettering Cancer Center, New York, NY USA; 6 Icahn School of Medicine at Mount Sinai, New York, NY USA; 7 Vejle Hospital and University of Southern Denmark, Vejle, Denmark; 8 Department of Lymphoma & Myeloma, The University of Texas MD Anderson Cancer Center, Houston, TX USA; 9 Arnie Charbonneau Cancer Research Institute, University of Calgary, Calgary, AB Canada; 10 Royal Wolverhampton NHS Trust and University of Wolverhampton, CRN West Midlands, NIHR, Wolverhampton, UK; 11 Department of Hematology, Hôpital Haut Lévêque, University Hospital, Pessac, France; 12 University of Melbourne, St Vincent’s Hospital, Melbourne, VIC Australia; 13 Department of Medicine/Haematology, NUI, Galway, Republic of Ireland; 14 CHU de Toulouse, IUCT-O, Université de Toulouse, UPS, Service d’Hématologie, Toulouse, France; 15 Department of Hematology, University Hospital, Nancy, France; 16 Department of Medical Oncology, Cross Cancer Institute, University of Alberta, Edmonton, AB Canada; 17 BC Cancer – Vancouver Centre Group, Vancouver, BC Canada; 18 Center for Multiple Myeloma, Massachusetts General Hospital Cancer Center, Boston, MA USA; 19 CHD Vendée, La Roche sur Yon, France; 20 Centre Hospitalier Universitaire (CHU) de Caen, Caen, France; 21 Hôpital Necker-Enfants Malades, Paris, France; 22 CHU Poitiers, Hôpital la Milétrie, Poitiers, France; 23 Cancer Research UK Clinical Trials Unit, Leeds Institute of Clinical Trials Research, University of Leeds, Leeds, UK; 24 Janssen Research & Development, LLC, Spring House, PA USA; 25 Janssen Research & Development, LLC, Titusville, NJ USA; 26 Janssen Research & Development, LLC, Raritan, NJ USA; 27 Department of Hematology, Mayo Clinic Rochester, Rochester, MN USA

**Keywords:** Targeted therapies, Myeloma

## Abstract

In the MAIA study, daratumumab plus lenalidomide and dexamethasone (D-Rd) improved progression-free survival (PFS) and overall survival (OS) versus lenalidomide and dexamethasone (Rd) alone in transplant-ineligible patients with newly diagnosed multiple myeloma (NDMM). We report updated efficacy and safety from MAIA (median follow-up, 64.5 months), including a subgroup analysis by patient age (<70, ≥70 to <75, ≥75, and ≥80 years). Overall, 737 transplant-ineligible patients with NDMM were randomized 1:1 to D-Rd or Rd. The primary endpoint, PFS, was improved with D-Rd versus Rd (median, 61.9 vs 34.4 months; hazard ratio [HR], 0.55; 95% confidence interval [CI], 0.45–0.67; *P* < 0.0001). Median OS was not reached in the D-Rd group versus 65.5 months in the Rd group (HR, 0.66; 95% CI, 0.53–0.83; *P* = 0.0003); estimated 60-month OS rates were 66.6% and 53.6%, respectively. D-Rd achieved higher rates of complete response or better (≥CR; 51.1% vs 30.1%), minimal residual disease (MRD) negativity (32.1% vs 11.1%), and sustained MRD negativity (≥18 months: 16.8% vs 3.3%) versus Rd (all *P* < 0.0001). D-Rd demonstrated clinically meaningful efficacy benefits across age groups. No new safety concerns were observed. Updated results (median follow-up, >5 years) continue to support frontline use of D-Rd in transplant-ineligible patients with NDMM.

## Introduction

Daratumumab is a human IgGκ monoclonal antibody targeting CD38 with a direct on-tumor [1–4] and immunomodulatory [5–7] mechanism of action, demonstrating greater cytotoxicity toward multiple myeloma (MM) cells ex vivo compared to analogs of other CD38 antibodies [8]. Daratumumab is approved as monotherapy for relapsed or refractory MM (RRMM) and in combination with standard-of-care regimens for RRMM and newly diagnosed MM (NDMM) [9, 10]. Several phase 3 studies have demonstrated an overall survival (OS) benefit with the addition of daratumumab to standard of care in both the RRMM and NDMM treatment settings [11–14].

The phase 3 MAIA study evaluated the efficacy and safety of daratumumab plus lenalidomide and dexamethasone (D-Rd) versus lenalidomide and dexamethasone (Rd) alone in transplant-ineligible patients with NDMM. In the primary analysis of MAIA, at a median follow-up of 28.0 months, D-Rd significantly improved progression-free survival (PFS; median, not reached vs 31.9 months; hazard ratio [HR], 0.56; 95% confidence interval [CI], 0.43–0.73; *P* < 0.001) and achieved a >2-fold increase in the rate of minimal residual disease (MRD) negativity versus Rd alone (24.2% vs 7.3%; *P* < 0.001) [15]. With longer follow-up (median follow-up, 56.2 months), D-Rd significantly improved OS versus Rd (median, not reached in either group; HR, 0.68; 95% CI, 0.53–0.86; *P* = 0.0013) [13]. No new safety concerns were reported with D-Rd treatment [13].

Subsequent analyses of MAIA evaluated sustained MRD negativity to determine its prognostic value in patients with transplant-ineligible NDMM [16]. With a median follow-up of 36.4 months, PFS was improved for patients who achieved MRD negativity and sustained MRD negativity lasting ≥6 and ≥12 months, regardless of treatment group. However, D-Rd was associated with higher rates of sustained MRD negativity lasting ≥6 months (14.9% vs 4.3%; nominal *P* < 0.0001) and ≥12 months (10.9% vs 2.4%; nominal *P* < 0.0001) versus Rd. Furthermore, a pooled analysis of MAIA and ALCYONE (a phase 3 study of daratumumab plus bortezomib, melphalan, and prednisone [D-VMP] versus bortezomib, melphalan, and prednisone [VMP] in transplant-ineligible patients with NDMM) showed that sustained MRD negativity lasting ≥6 or ≥12 months was associated with improved PFS, which was supported by both univariate and multivariate Cox regression analyses [16].

In July 2021, a protocol amendment was implemented for the MAIA study, leading to a long-term extension (89.3-month median follow-up) where data capture in the clinical database was limited to subsequent therapies, survival follow-up, death information, end-of-treatment disposition, and end-of-study disposition. These findings were previously presented [17]. Here, we report results from an updated analysis of the efficacy and safety in MAIA at a median follow-up of 64.5 months, which was the final longest follow-up of the MAIA trial that comprised a complete dataset. We also report results from a subgroup analysis of patients from MAIA based on age (<70 years, ≥70 to <75 years, ≥75 years, and ≥80 years).

## Methods

### Trial design and oversight

MAIA (ClinicalTrials.gov Identifier: NCT02252172) is a multicenter, randomized, open-label, phase 3 study in transplant-ineligible patients with NDMM. The study design has been published previously [15]. Briefly, eligible patients had documented NDMM, an Eastern Cooperative Oncology Group performance status of 0 to 2, and were ineligible for high-dose chemotherapy with autologous stem cell transplant because of age (≥65 years) or comorbidities.

### Ethics approval and consent to participate

The independent ethics committee or institutional review board approved the protocol at each site (Supplementary Table 1). All methods were performed in accordance with the relevant guidelines and regulations. The trial was conducted in accordance with the principles of the Declaration of Helsinki and the International Conference on Harmonisation Good Clinical Practice guidelines. All patients provided written informed consent.

### Randomization and study treatment

Patients were randomly assigned 1:1 to treatment with D-Rd or Rd and were stratified by International Staging System disease stage (I vs II vs III), geographic region (North America vs other), and age (<75 vs ≥75 years). All patients received 28-day cycles of oral lenalidomide (25 mg [10 mg if the patient’s creatinine clearance was 30–50 mL/min] on Days 1–21) and oral dexamethasone (40 mg [20 mg if the patient was aged >75 years or had a body mass index <18.5 kg/m^2^] on Days 1, 8, 15, and 22) until disease progression or unacceptable toxicity. Patients in the D-Rd group also received intravenous daratumumab 16 mg/kg once weekly during Cycles 1 and 2, every 2 weeks during Cycles 3 through 6, and every 4 weeks thereafter.

### Endpoints and assessments

PFS was the primary endpoint, defined as the time from randomization to disease progression or death, whichever occurred first. Secondary endpoints included complete response (CR) rate, stringent CR rate, overall response rate (ORR), MRD-negativity rate, PFS on next line of therapy, and OS. Response and disease progression were assessed based on International Myeloma Working Group criteria [18, 19] using a validated computer algorithm. MRD was assessed (at baseline, at the time of suspected ≥CR, and at prespecified intervals [12, 18, 24, 30, 36, 48, and 60 months after the first dose] in patients with a near ≥CR) using bone marrow aspirate samples and was evaluated via next-generation sequencing using the clonoSEQ® assay (v.2.0; Adaptive Biotechnologies, Seattle, WA, USA). PFS on next line of therapy was defined as the time from randomization to progression on next line of therapy or death, whichever occurred first. Patients were censored at the last disease assessment before starting their next line of therapy if they did not show disease progression on study treatment. Patients who started their next line of therapy after progressing on study treatment, were still alive, and had not yet progressed on next line of therapy were censored on the last date of follow-up. Patients without post-baseline follow-up were censored at randomization.

### Statistical analyses

Statistical methods have been published previously [13, 15]. The primary analysis was performed in the intent-to-treat (ITT) population, which included all patients who underwent randomization. PFS and OS were compared between treatment groups using a log-rank test stratified with randomization stratification factors. The Kaplan–Meier method was used to estimate distributions; HRs and 95% CIs were estimated using a Cox regression model, with treatment as the sole explanatory variable and stratified with randomization stratification factors. Response rates were compared between treatment groups using the Cochran–Mantel–Haenszel chi-square test. For age and other subgroup analyses, PFS, OS, and response rates were analyzed similarly to the overall ITT population, but randomization stratification factors were excluded. MRD-negativity rates were compared between treatment groups using the Fisher’s exact test. Nominal *P*-values were calculated for all analyses reported herein. Safety analyses were performed in the safety population, which included all randomized patients who received ≥1 dose of study treatment.

### Role of funding source

The study sponsor funded this study and, in collaboration with the authors, designed the trial and collected, analyzed, and interpreted the data. All authors had full access to all of the data in the study and had responsibility for the decision to submit for publication.

### Data sharing statement

The data sharing policy of Janssen Pharmaceutical Companies of Johnson & Johnson is available at https://www.janssen.com/clinical-trials/transparency. As noted on this site, requests for access to the study data can be submitted through Yale Open Data Access (YODA) Project site at http://yoda.yale.edu.

## Results

### Patients

A total of 737 patients (D-Rd, *n* = 368; Rd, *n* = 369) were randomized in MAIA. Baseline characteristics were well balanced between treatment arms (Supplementary Table 2) [15]. Baseline demographics and clinical characteristics by age subgroup (<70, ≥70 to <75, ≥75, and ≥80 years) are shown in Supplementary Table 2. Median treatment duration was 47.5 months with D-Rd and 22.6 months with Rd. Treatment exposure for the safety population is summarized in Supplementary Table 3. At the time of clinical data cutoff for this updated analysis (October 21, 2021), a total of 233 (64.0%) patients in the D-Rd group and 311 (85.2%) patients in the Rd group discontinued treatment; the most common reasons for treatment discontinuation were disease progression (D-Rd, 29.4%; Rd, 35.9%) and adverse events (AEs; D-Rd, 15.7%; Rd, 24.4%). The proportion of patients who discontinued treatment was lower in the D-Rd arm versus the Rd arm across age subgroups (Supplementary Table 4).

### Efficacy

**Fig. 1:**
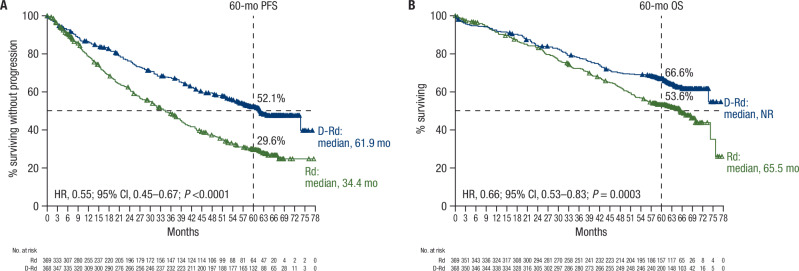
PFS and OS in the ITT population. Kaplan–Meier estimates of (**A**) PFS and (**B**) OS in the ITT population. PFS progression-free survival, OS overall survival, ITT intent-to-treat, D-Rd daratumumab plus lenalidomide/dexamethasone, Rd lenalidomide/dexamethasone, HR hazard ratio, CI confidence interval, NR not reached.

**Fig. 2:**
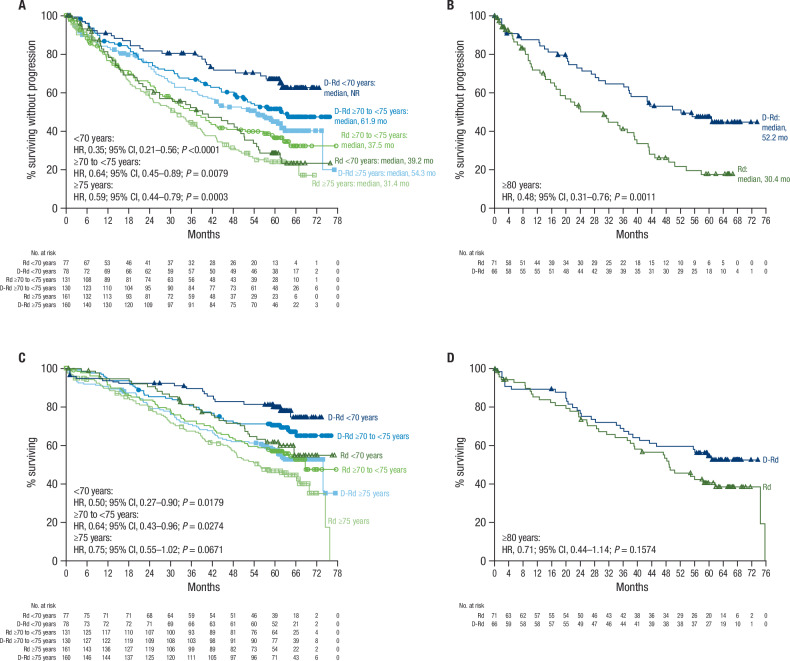
PFS and OS by age subgroup. Kaplan–Meier estimates of (**A**) PFS in patients aged <70 years, ≥70 to <75 years, and ≥75 years; (**B**) PFS in patients aged ≥80 years; (**C**) OS in patients aged <70 years, ≥70 to <75 years, and ≥75 years; and (**D**) OS in patients aged ≥80 years. PFS progression-free survival, OS overall survival, D-Rd daratumumab plus lenalidomide/dexamethasone, Rd lenalidomide/dexamethasone, HR hazard ratio, CI confidence interval, NR not reached.

At a median follow-up of 64.5 months (range, 0–77.6), median PFS was 61.9 months with D-Rd versus 34.4 months with Rd in the ITT population (HR, 0.55; 95% CI, 0.45–0.67; *P* < 0.0001); estimated 60-month PFS rates were 52.1% and 29.6%, respectively (Fig. 1A). Among patients with confirmed disease progression in each group, the most common reason for disease progression was biochemical (D-Rd, 76.2%; Rd, 83.6%), followed by bone lesion (D-Rd, 16.4%; Rd, 13.6%), plasmacytoma (D-Rd, 8.2%; Rd, 4.5%), and other (D-Rd, 1.6%; Rd, 2.3%). PFS was improved with D-Rd versus Rd in subgroups of patients aged <70 (HR, 0.35; 95% CI, 0.21–0.56; *P* < 0.0001), ≥70 to <75 (HR, 0.64; 95% CI, 0.45–0.89; *P* = 0.0079), and ≥75 years (HR, 0.59; 95% CI, 0.44–0.79; *P* = 0.0003; Fig. 2A), as well as in patients aged ≥80 years (HR, 0.48; 95% CI, 0.31–0.76; *P* = 0.0011; Fig. 2B). The PFS benefit with D-Rd versus Rd was consistent across prespecified subgroups by baseline characteristics (Supplementary Fig. 1).

Median OS was not reached in the D-Rd arm versus 65.5 months in the Rd arm (HR, 0.66; 95% CI, 0.53–0.83; *P* = 0.0003); estimated 60-month OS rates were 66.6% and 53.6%, respectively (Fig. 1B). OS was improved with D-Rd versus Rd in subgroups of patients aged <70 (HR, 0.50; 95% CI, 0.27–0.90; *P* = 0.0179), ≥70 to <75 (HR, 0.64; 95% CI, 0.43–0.96; *P* = 0.0274), and ≥75 years (HR, 0.75; 95% CI, 0.55–1.02; *P* = 0.0671; Fig. 2C), as well as in patients aged ≥80 years (HR, 0.71; 95% CI, 0.44–1.14; *P* = 0.1574; Fig. 2D). OS benefit with D-Rd versus Rd was generally consistent across prespecified patient subgroups by baseline characteristics (Supplementary Fig. 2).

A total of 52 patients in the D-Rd group discontinued lenalidomide ± dexamethasone and remained on the rest of study treatment at clinical cutoff; 46 (88.5%) patients discontinued due to AEs (Supplementary Table 5). Among these 52 patients in the D-Rd arm who discontinued only lenalidomide ± dexamethasone, the median time to lenalidomide discontinuation was 37.8 months (range, 1–70), and median daratumumab treatment duration was 66.2 months (range, 56–77). Estimated 60-month PFS and OS rates for the 52 patients in the D-Rd arm who discontinued lenalidomide ± dexamethasone but continued remaining treatment were 98.1% and 100.0%, respectively. Thirteen (25.0%) of the 52 patients discontinued lenalidomide and continued on daratumumab + dexamethasone, and 39 (75.0%) of the 52 patients discontinued lenalidomide + dexamethasone and continued on daratumumab monotherapy. Among the 39 patients who discontinued lenalidomide + dexamethasone and continued on daratumumab monotherapy, median time to lenalidomide + dexamethasone discontinuation was 39.1 months (range, 3–67), and median daratumumab treatment duration was 65.6 months (range, 56–73). Estimated 60-month PFS and OS rates for the 39 patients in the D-Rd arm who discontinued lenalidomide +dexamethasone but continued on daratumumab monotherapy were 97.4% and 100.0%, respectively. One patient in the D-Rd group discontinued daratumumab but remained on lenalidomide treatment at clinical cutoff; the patient discontinued daratumumab after 15 days of treatment due to AEs. The patient had not progressed at the time of clinical cutoff.

**Fig. 3:**
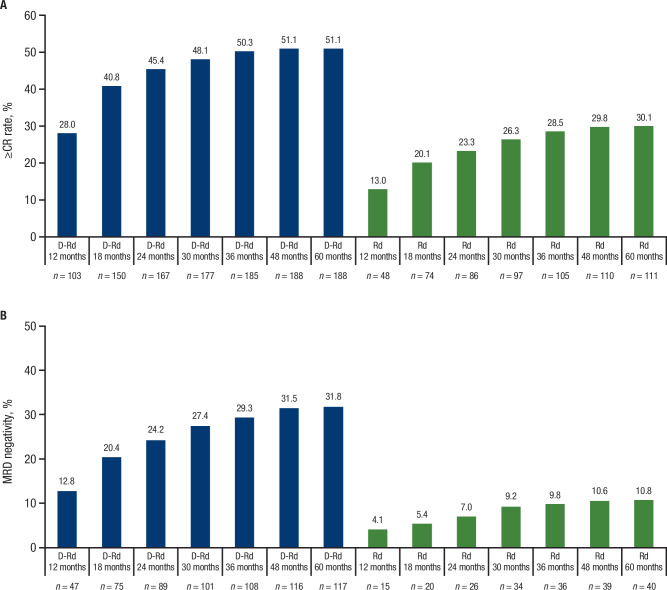
Cumulative best response rates of ≥CR and MRD negativity over time in the ITT population. **A** Cumulative ≥CR and (**B**) MRD-negativity rates by 12, 18, 24, 30, 36, 48, and 60 months in the ITT population. CR complete response, MRD minimal residual disease, ITT intent-to-treat, D-Rd daratumumab plus lenalidomide/dexamethasone, Rd lenalidomide/dexamethasone.

**Fig. 4:**
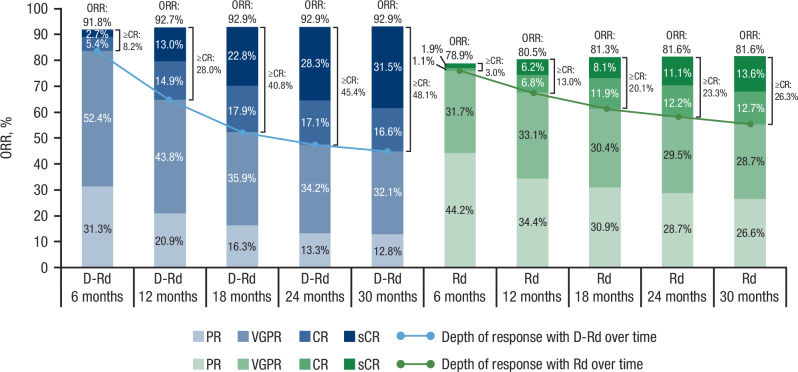
Response rates over time in the ITT population. Response rates by 6, 12, 18, 24, and 30 months in the ITT population. ITT intent-to-treat, ORR overall response rate, CR complete response, D-Rd daratumumab plus lenalidomide/dexamethasone, Rd lenalidomide/dexamethasone, PR partial response, VGPR very good partial response, sCR stringent complete response.

The ORR (92.9% vs 81.6%) and rates of CR or better (≥CR; 51.1% vs 30.1%) and very good partial response or better (≥VGPR; 81.5% vs 56.9%) were significantly higher with D-Rd versus Rd in the ITT population (all *P* < 0.0001; Table 1). ORRs were also higher for D-Rd versus Rd across all age subgroups, as were rates of ≥CR and ≥VGPR (Table 2). Cumulative best response rates improved with continuous D-Rd treatment in patients who achieved ≥CR (Fig. 3A and Supplementary Fig. 3A) and those who achieved ≥VGPR (Supplementary Fig. 3B). Best response rates markedly deepened over time with continued D-Rd treatment, with ≥CR rates increasing from 8.2% by 6 months to 28.0% by 12 months, 40.8% by 18 months, 45.4% by 24 months, 48.1% by 30 months, and 51.1% by 48 months (Figs. 3A and 4, and Supplementary Fig. 3A). In patients who achieved ≥CR, median PFS was not reached in either treatment arm (HR, 0.52; 95% CI, 0.35–0.76; *P* = 0.0007); estimated 60-month PFS rates were 73.7% with D-Rd and 53.8% with Rd. In patients who achieved VGPR, median PFS was 42.7 months with D-Rd versus 36.2 months with Rd (HR, 0.71; 95% CI, 0.51–0.99; *P* = 0.0401); estimated 60-month PFS rates were 37.1% versus 23.2%, respectively. Median OS was not reached for patients who achieved ≥CR in either treatment arm (HR, 0.58; 95% CI, 0.37–0.91; *P* = 0.0164); estimated 60-month OS rates were 81.7% with D-Rd versus 69.1% with Rd. Median OS was not reached with D-Rd versus 63.1 months with Rd for patients who achieved VGPR (HR, 0.78; 95% CI, 0.53–1.16; *P* = 0.2256); estimated 60-month OS rates were 61.5% versus 53.0%, respectively.

**Table 1:** Response rates and MRD-negativity rates in the ITT population.

	D-Rd (*n* = 368)	Rd (*n* = 369)	*P* value
Response, *n* (%)			
ORR	342 (92.9)	301 (81.6)	< 0.0001^a^
≥CR	188 (51.1)	111 (30.1)	< 0.0001^a^
sCR	131 (35.6)	58 (15.7)	< 0.0001^a^
CR	57 (15.5)	53 (14.4)	
≥VGPR	300 (81.5)	210 (56.9)	< 0.0001^a^
VGPR	112 (30.4)	99 (26.8)	
PR	42 (11.4)	91 (24.7)	
SD	11 (3.0)	55 (14.9)	
PD	1 (0.3)	0	
NE	14 (3.8)	13 (3.5)	
MRD negative (10^–5^), *n* (%)	118 (32.1)	41 (11.1)	< 0.0001^b^
Sustained MRD negative (10^–5^), *n* (%)			
Lasting ≥12 months	69 (18.8)	15 (4.1)	< 0.0001^b^
Lasting ≥18 months	62 (16.8)	12 (3.3)	< 0.0001^b^

*MRD* minimal residual disease, *ITT* intent-to-treat, *D-Rd* daratumumab plus lenalidomide/dexamethasone, *Rd* lenalidomide/dexamethasone, *ORR* overall response rate, *CR* complete response, *sCR* stringent complete response, *VGPR* very good partial response, *PR* partial response, *SD* stable disease, *PD* progressive disease, *NE* not evaluable.

^a^*P* value was calculated using the Cochran–Mantel–Haenszel chi-square test.

^b^*P* value was calculated using the Fisher’s exact test.

The rate of MRD negativity (10^–5^ sensitivity) was significantly higher for D-Rd versus Rd (32.1% vs 11.1%), as were rates of sustained MRD negativity lasting for ≥12 months (18.8% vs 4.1%) and ≥18 months (16.8% vs 3.3%; all *P* < 0.0001; Table 1). Responses also deepened over time with respect to MRD negativity; MRD-negativity rates increased from 12.8% at 12 months to 20.4% at 18 months, 24.2% at 24 months, 27.4% at 30 months, 29.3% at 36 months, 31.5% at 48 months, and 31.8% at 60 months (Fig. 3B). Increased rates of MRD negativity were observed with D-Rd versus Rd across all age subgroups (Table 2). PFS and OS were improved in patients who achieved MRD negativity versus patients who were MRD positive in both treatment arms; however, more patients in the D-Rd arm achieved MRD negativity (Supplementary Fig. 4A, B).

**Table 2:** Response rates and MRD-negativity rates by age subgroup.

	< 70 years			≥70 to < 75 years			≥75 years			≥80 years		
	D-Rd (*n* = 78)	Rd (*n* = 77)	*P* value	D-Rd (*n* = 130)	Rd (*n* = 131)	*P* value	D-Rd (*n* = 160)	Rd (*n* = 161)	*P* value	D-Rd (*n* = 66)	Rd (*n* = 71)	*P* value
Response, *n* (%)												
ORR	73 (93.6)	62 (80.5)	0.0156^a^	125 (96.2)	108 (82.4)	0.0004^a^	144 (90.0)	131 (81.4)	0.0275^a^	59 (89.4)	55 (77.5)	0.0629^a^
≥CR	44 (56.4)	24 (31.2)	0.0016^a^	73 (56.2)	41 (31.3)	< 0.0001^a^	71 (44.4)	46 (28.6)	0.0033^a^	29 (43.9)	15 (21.1)	0.0044^a^
sCR	31 (39.7)	11 (14.3)	0.0004^a^	50 (38.5)	23 (17.6)	0.0002^a^	50 (31.3)	24 (14.9)	0.0005	23 (34.8)	8 (11.3)	0.0010^a^
CR	13 (16.7)	13 (16.9)		23 (17.7)	18 (13.7)		21 (13.1)	22 (13.7)		6 (9.1)	7 (9.9)	
≥VGPR	64 (82.1)	45 (58.4)	0.0013^a^	111 (85.4)	76 (58.0)	< 0.0001^a^	125 (78.1)	89 (55.3)	< 0.0001^a^	50 (75.8)	31 (43.7)	0.0001^a^
VGPR	20 (25.6)	21 (27.3)		38 (29.2)	35 (26.7)		54 (33.8)	43 (26.7)		21 (31.8)	16 (22.5)	
PR	9 (11.5)	17 (22.1)		14 (10.8)	32 (24.4)		19 (11.9)	42 (26.1)		9 (13.6)	24 (33.8)	
SD	1 (1.3)	14 (18.2)		3 (2.3)	20 (15.3)		7 (4.4)	21 (13.0)		3 (4.5)	11 (15.5)	
PD	0	0		0	0		1 (0.6)	0		0	0	
NE	4 (5.1)	1 (1.3)		2 (1.5)	3 (2.3)		8 (5.0)	9 (5.6)		4 (6.1)	5 (7.0)	
MRD negative (10^–5^), *n* (%)	28 (35.9)	9 (11.7)	0.0006^b^	47 (36.2)	16 (12.2)	< 0.0001^b^	43 (26.9)	16 (9.9)	< 0.0001^b^	17 (25.8)	4 (5.6)	0.0016^b^

*MRD* minimal residual disease, *D-Rd* daratumumab plus lenalidomide/dexamethasone, *Rd* lenalidomide/dexamethasone, *ORR* overall response rate, *CR* complete response, *sCR* stringent complete response, *VGPR* very good partial response, *PR* partial response, *SD* stable disease, *PD* progressive disease, *NE* not evaluable.

^a^*P* value was calculated using the Cochran–Mantel–Haenszel chi-square test.

^b^*P* value was calculated using the Fisher’s exact test.

At clinical data cutoff, 128 (35.2%) patients in the D-Rd arm and 194 (53.2%) patients in the Rd arm had received subsequent therapy. Among those who received subsequent therapy, 9.4% of patients in the D-Rd arm and 23.2% of patients in the Rd arm received a daratumumab-containing treatment as their first subsequent therapy, and 14.1% and 48.5%, respectively, received a daratumumab-containing treatment in any subsequent line of therapy. PFS on next line of therapy was 73.7 months in the D-Rd arm versus 48.9 months in the Rd arm (HR, 0.61; 95% CI, 0.49–0.76; *P* < 0.0001).

### Safety

No new safety concerns were observed with longer follow-up (Table 3). Grade 3/4 treatment-emergent AEs (TEAEs) were reported in 95.9% of patients in the D-Rd arm and 88.8% of patients in the Rd arm. The most common (≥20%) grade 3/4 TEAEs with D-Rd versus Rd were neutropenia (54.1% vs 37.0%) and anemia (17.0% vs 21.6%). Grade 3/4 infection rates were 42.6% with D-Rd and 29.6% with Rd. Serious TEAEs were reported in 78.8% of patients in the D-Rd arm and 71.0% of patients in the Rd arm, with pneumonia being the most common serious TEAE (18.7% and 10.7%, respectively). Overall rate of study treatment discontinuation due to TEAEs was lower with D-Rd versus Rd (14.6% vs 23.8%, respectively). TEAEs led to discontinuation of lenalidomide in 36.8% of patients in the D-Rd arm and 24.4% of patients in the Rd arm, and TEAEs led to discontinuation of dexamethasone in 39.8% of patients in the D-Rd arm and 36.2% of patients in the Rd arm. Rate of daratumumab discontinuation due to TEAEs was 14.6%. TEAEs with an outcome of death occurred in 9.9% of patients in the D-Rd arm and 9.3% of patients in the Rd arm.

**Table 3:** Most common any grade or grade 3/4 TEAEs in the safety population.

	D-Rd (*n* = 364)		Rd (*n* = 365)	
TEAE, *n* (%)	Any grade	Grade 3/4	Any grade	Grade 3/4
Hematologic				
Neutropenia	224 (61.5)	197 (54.1)	166 (45.5)	135 (37.0)
Anemia	154 (42.3)	62 (17.0)	150 (41.1)	79 (21.6)
Nonhematologic				
Diarrhea	240 (65.9)	33 (9.1)	188 (51.5)	22 (6.0)
Fatigue	164 (45.1)	33 (9.1)	114 (31.2)	17 (4.7)
Constipation	157 (43.1)	6 (1.6)	137 (37.5)	2 (0.5)
Peripheral edema	155 (42.6)	10 (2.7)	117 (32.1)	3 (0.8)
Back pain	155 (42.6)	14 (3.8)	109 (29.9)	14 (3.8)
Asthenia	136 (37.4)	19 (5.2)	101 (27.7)	18 (4.9)
Nausea	133 (36.5)	7 (1.9)	88 (24.1)	2 (0.5)
Insomnia	125 (34.3)	11 (3.0)	116 (31.8)	14 (3.8)
Bronchitis	124 (34.1)	12 (3.3)	87 (23.8)	7 (1.9)
Cough	123 (33.8)	2 (0.5)	65 (17.8)	0
Dyspnea	119 (32.7)	12 (3.3)	63 (17.3)	4 (1.1)
Pneumonia	113 (31.0)	71 (19.5)	66 (18.1)	39 (10.7)
Weight decreased	112 (30.8)	10 (2.7)	69 (18.9)	11 (3.0)
Peripheral sensory neuropathy	111 (30.5)	9 (2.5)	66 (18.1)	2 (0.5)
Muscle spasms	111 (30.5)	2 (0.5)	86 (23.6)	5 (1.4)

Any grade TEAEs and grade 3/4 TEAEs that are listed are those that occurred in ≥30% and ≥20% of patients in either group, respectively.

*TEAE* treatment-emergent adverse event, *D-Rd* daratumumab plus lenalidomide/dexamethasone, *Rd* lenalidomide/dexamethasone.

Grade 3/4 TEAEs occurred in 95.5% of patients in the D-Rd arm and 95.0% of those in the Rd arm who were aged ≥75 years and in 92.3% and 95.7%, respectively, who were aged ≥80 years. Rates of common grade 3/4 TEAEs in patients aged ≥75 years and ≥80 years were generally similar to those of the overall study population (Supplementary Table 6). Among patients aged ≥75 years, serious TEAEs occurred in 80.9% of patients in the D-Rd arm and 79.2% of those in the Rd arm, the most common of which was pneumonia (19.7% and 12.6%, respectively). Additionally, for patients aged ≥75 years, TEAEs led to study treatment discontinuation in 15.3% of patients in the D-Rd arm and 27.7% of those in the Rd arm, and TEAEs with an outcome of death occurred in 11.5% and 13.2% of patients, respectively. Among patients aged ≥80 years, serious TEAEs occurred in 81.5% of patients in the D-Rd arm and 82.9% of those in the Rd arm, with pneumonia being most common (24.6% and 8.6%, respectively). Additionally, for patients aged ≥80 years, TEAEs led to study treatment discontinuation in 6.2% of patients in the D-Rd arm and 20.0% of those in the Rd arm, and TEAEs with an outcome of death occurred in 12.3% and 11.4% of patients, respectively.

## Discussion

This updated analysis of MAIA, with a median follow-up of 64.5 months, confirmed results of the primary efficacy analysis [15] and the interim OS analysis [13], which demonstrated statistically significant and clinically meaningful improvement in PFS and OS with D-Rd versus Rd treatment until disease progression in transplant-ineligible patients with NDMM. Treatment with D-Rd resulted in a 45% reduction in the risk of disease progression or death versus Rd. Moreover, a 34% reduction in the risk of death was observed with D-Rd. These clinically meaningful improvements are particularly noteworthy given the lower median relative dose intensity of lenalidomide in the D-Rd arm. PFS and OS benefits with D-Rd versus Rd were generally consistent across prespecified patient subgroups. Deepening of response continued over time for patients who remained on D-Rd treatment, as best response of ≥CR rates with D-Rd more than tripled from 6 months (8.2%) to 12 months (28.0%) and continued to increase with continued therapy up to 51.1% by 48 months. Patients who achieved a best response of ≥CR had higher 60-month PFS rates (73.7% with D-Rd vs 53.8% with Rd) and 60-month OS rates (81.7% with D-Rd vs 69.1% with Rd) than patients who achieved a best response of VGPR (37.1% vs 23.2% and 61.5% vs 53.0%, respectively). D-Rd also achieved a nearly 2-fold higher MRD-negativity rate and >3-fold higher sustained MRD-negativity rates lasting ≥12 and ≥18 months versus Rd alone. MRD-negativity rates also increased over time with continued D-Rd treatment, more than doubling from 12 months (12.8%) to 60 months (31.8%). Patients who achieved MRD negativity had improved PFS and OS versus those who were MRD positive. Thus, patients who continued on D-Rd therapy had an increased likelihood of achieving deeper responses, translating to improved long-term outcomes.

Results from a subgroup analysis of patients from MAIA based on age (<70, ≥70 to <75, ≥75, and ≥80 years) were consistent with those reported for the ITT population. D-Rd demonstrated a meaningful benefit versus Rd in clinically relevant endpoints, including PFS, OS, ORR, and MRD-negativity rate, across age subgroups. Moreover, D-Rd induced higher rates of deep responses versus Rd across all age subgroups evaluated.

No new safety concerns were observed with longer follow-up. While the rates of grade 3/4 TEAEs and serious TEAEs were higher with D-Rd versus Rd, the rate of study treatment discontinuation due to TEAEs was lower with D-Rd versus Rd, the latter of which may primarily be attributable to the MAIA study design. Specifically, in the Rd group, lenalidomide discontinuation was categorized as overall study treatment discontinuation, whereas, in the D-Rd group, overall study treatment discontinuation required discontinuation of both daratumumab and lenalidomide. In subgroup analyses of safety in patients aged ≥75 and ≥80 years, the rates of grade 3/4 TEAEs and serious TEAEs were similar for D-Rd and Rd, and the rate of study treatment discontinuation due to TEAEs was lower for D-Rd versus Rd.

The benefits observed with D-Rd treatment until disease progression in MAIA were consistent with those observed in the phase 3 FIRST study, in which Rd treatment until disease progression improved PFS and achieved more durable responses than 18 cycles of Rd in transplant-ineligible patients with NDMM [20]. In a post hoc analysis of the FIRST trial, the cumulative ≥VGPR rate with continuous Rd treatment continued to increase beyond 18 months of treatment, and the benefit of this regimen was more profound in patients achieving CR versus patients achieving ≥VGPR or partial response or better [21]. This is consistent with the MAIA data, showing higher 60-month PFS and OS rates in patients who achieved a best response of ≥CR than in those who achieved VGPR. In a pooled analysis of patients with high-risk cytogenetic abnormalities from MAIA and the phase 3 ALCYONE study, achievement of ≥CR with daratumumab was associated with improved PFS, demonstrating the importance of achieving ≥CR to improve treatment outcomes [22].

While cross-trial comparisons should be interpreted with caution, it is noteworthy that the MAIA results compare favorably to those of the phase 3 SWOG S0777 study of bortezomib plus Rd (VRd) versus Rd alone in patients with NDMM without intent for immediate transplant. At a median follow-up of 84 months, the median PFS was 41 months for VRd and 29 months for Rd (stratified HR, 0.742; 96% Wald CI, 0.594–0.928; 1-sided stratified log-rank *P* = 0.003), and median OS was not reached for VRd and 69 months for Rd (stratified HR, 0.709; 96% Wald CI, 0.543–0.926; 2-sided stratified *P* = 0.0114) [23]. No significant improvement in OS was observed with VRd versus Rd in the subgroup of patients aged ≥65 years (median, 65 vs 56 months; HR, 0.769; 2-sided stratified *P* = 0.168). Importantly, median age was higher in the MAIA study than in SWOG S0777 (73 vs 63 years), more patients in MAIA were aged ≥65 years than in SWOG S0777 (99% vs 43%), and all patients in MAIA were ineligible for transplant, while 32% of SWOG S0777 patients were not intended for future transplant [13, 23, 24]. In addition, patients in MAIA received D-Rd triplet therapy until disease progression, whereas patients in SWOG S0777 received 8 cycles of VRd triplet therapy followed by Rd until disease progression.

Evidence from the current analysis further strengthens previous results [16] demonstrating the prognostic value of MRD negativity. With longer median follow-up of MAIA (>5 years), responses continued to deepen over time with continued D-Rd therapy, including rates of ≥CR and MRD negativity, leading to long-term clinically meaningful benefits in PFS and OS. Rates of ≥CR were significantly higher with D-Rd versus Rd, and patients who achieved ≥CR had higher 60-month PFS and OS rates than patients who achieved VGPR. Moreover, higher rates of MRD negativity and sustained MRD negativity lasting ≥12 and ≥18 months were observed with D-Rd versus Rd. While patients who achieved MRD negativity had improved PFS and OS versus those who were MRD positive, D-Rd treatment further improved PFS and OS outcomes in patients who achieved MRD negativity. These results, combined with results of previous analyses [16], suggest that MRD status may serve as a more accurate prognostic indicator for clinical outcomes than relying on hematologic response rates (ie, CR) alone. As MRD negativity may serve as a more robust measure of disease control if it is sustained over time, sustained MRD negativity lasting ≥6 months, ≥12 months, and beyond may represent yet an even deeper response with a greater prognostic value.

A limitation of this study is that the results focus on outcomes of patients treated with D-Rd versus Rd in their first line of therapy. Among patients in the Rd group who received subsequent therapy, 23% of patients received a daratumumab-containing regimen as first subsequent therapy and 49% received a daratumumab-containing regimen as any subsequent line of therapy. Despite this degree of crossover, the D-Rd group demonstrated a significant survival advantage over the Rd group, underscoring the importance of using D-Rd as first-line treatment in this setting and identifying potential barriers to accessing treatment. Limited access to daratumumab, shaped by factors such as patient location, might have led to fewer patients receiving a daratumumab-based regimen as subsequent therapy.

In conclusion, these updated efficacy and safety analyses from MAIA after a median follow-up of >5 years continue to support frontline use of D-Rd in transplant-ineligible patients with NDMM. These results, comprising the longest follow-up of MAIA with a complete dataset, together with the OS benefit observed with daratumumab-containing regimens in the ALCYONE, CASSIOPEIA, CASTOR, and POLLUX studies [11, 12, 14, 25] continue to support the use of daratumumab in patients with MM.

## Supplementary information

Supplementary Appendix
